# Systems integrity in health and aging - an animal model approach

**DOI:** 10.1186/2046-2395-2-2

**Published:** 2013-01-07

**Authors:** Marije Oostindjer, Gro V Amdam

**Affiliations:** 1 Department of Chemistry, Biotechnology and Food Science, Norwegian University of Life Sciences, PO Box 5003, N-1432, Aas, Norway; 2 School of Life Sciences, Arizona State University, PO Box 874501, 85287, Tempe, AZ, USA

## Abstract

Human lifespan is positively correlated with childhood intelligence, as measured by psychometric (IQ) tests. The strength of this correlation is similar to the negative effect that smoking has on the life course. This result suggests that people who perform well on psychometric tests in childhood may remain healthier and live longer. The correlation, however, is debated: is it caused exclusively by social-environmental factors or could it also have a biological component? Biological traits of systems integrity that might result in correlations between brain function and lifespan have been suggested but are not well-established, and it is questioned what useful knowledge can come from understanding such mechanisms. In a recent study, we found a positive correlation between brain function and longevity in honey bees. Honey bees are highly social, but relevant social-environmental factors that contribute to cognition-survival correlations in humans are largely absent from insect colonies. Our results, therefore, suggest a biological explanation for the correlation in the bee. Here, we argue that individual differences in stress handling (coping) mechanisms, which both affect the bees’ performance in tests of brain function and their survival could be a trait of systems integrity. Individual differences in coping are much studied in vertebrates, and several species provide attractive models. Here, we discuss how pigs are an interesting model for studying behavioural, physiological and molecular mechanisms that are recruited during stress and that can drive correlations between health, cognition and longevity traits. By revealing biological factors that make individuals susceptible to stress, it might be possible to alleviate health and longevity disparities in people.

## Background

As the demography of many countries shifts toward a larger proportion of elderly people, it has become a priority to understand how environmental factors and biological mechanisms contribute to differences in longevity and healthiness during aging [1-3]. This priority continues to grow stronger with the increasing economic burden of elder care on societies, and will increasingly demand that attention be placed on how good health and brain function can be achieved in the older cohorts [2].

The quality of human health during mid-life and old age differs between socioeconomic groups [1,4]. Low income and low social status correlate with more illnesses over the life course, disability during ageing, as well as death at younger ages [4-7]. This association can be explained by associations between socioeconomic status, education, workload, behaviour, diet, body weight and lifetime exposures to stressful or damaging environmental conditions [4,6-10]. All of these factors also correlate with childhood or young adult IQ that provides a unit of measure for intelligence and brain function [4,9,11-14]. Environmental factors that influence both cognitive ability and health in a negative way, and therefore can cause these traits to become correlated, include illnesses and degenerative effects of privations such as nutritional stress before or after birth [6,15]. High cognitive performance, reciprocally, correlates with positive educational and occupational life outcomes that can benefit health and longevity [6,12,16-18]. Cognition-survival correlations, thereby, appear to be strongly mediated by environmental factors.

Part of the covariance between cognitive test performance (such as IQ scores) and lifespan, however, is not well explained by environmental factors and may stem from lesser-known biological influences that impact people’s abilities to cope with lifetime events [19]. These biological influences may be gene-mediated, but are not limited to genetics: biological influences may be largely mediated by gene-environment interactions (for example prenatal stress). System integrity refers to traits that are part of the initial state of the system, and are suggested to include ‘functional reserve capacity’, which is the ability to maintain brain function during degenerative processes, and ‘metabolic robustness’, which is the ability to maintain metabolic stability despite induced metabolic stress [3,6,20]. These and other aspects of system integrity might explain some variation in successful ageing, and perhaps provide a basis for interventions that can increase healthspan and longevity [21]. Thus far, however, cognition-survival correlations are debated and poorly understood [3,22,23]. In this paper we focus on the potential importance of stress coping (the ability to adapt to stressful situations) as a trait of system integrity, and present two model animals that have complimentary advantages for the study of system integrity.

## Review

### The honey bee: a model for systems integrity?

**Figure 1:**
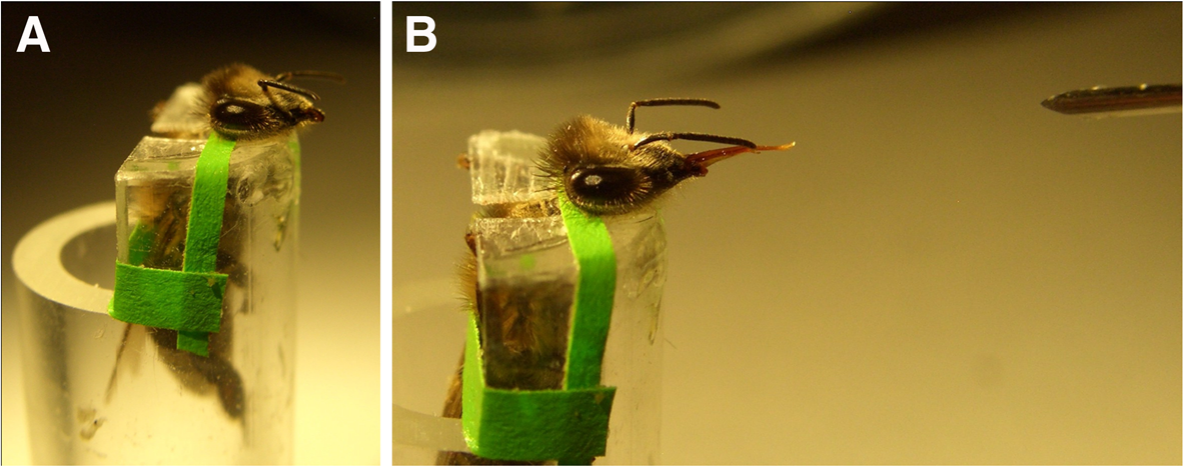
**Worker bee prepared for learning test.** The bee holder is custom-made in Plexiglas; the bee is secured with straps. After learning or memory retrieval, the straps are removed and the bee is released unharmed. In panel **A**, the bee rests between CS-US pairings. In panel **B**, the bee reveals learning by extending her tongue in a PER to the CS alone. CS is a flower odour (carnation) that is expelled from the syringe-tip to the far right in the image. CS, conditioned stimulus; PER, proboscis extension reflex; US, unconditioned stimulus.

In research on ageing, animals are used to model syndromes of human senescence with the hope that results will facilitate new strategies to improve elderly health and longevity [24]. For example, candidate longevity genes from the nematode *Caenorhabditis elegans* were recently used to identify genes that influence variance in cognitive ability and age-related cognitive decline in humans [25]. Another model with moderate complexity is ageing plasticity in honey bees [26]. In honey bees, variation in brain function and lifespan can be measured individually, complex environmental factors such as social influences can be controlled, and the availability of genome sequence with predicted genes and proteins [27] facilitates molecular research [28,29]. The majority of these experiments study ‘worker’ bees; an essentially sterile female caste that represents the majority of individuals in a honey bee colony. Worker bees are very amenable to experimental handling (for example Figure 1), and many aspects of their biology, including social environment and behaviours, workload, diet, learning, memory, communication, and ageing, can be manipulated with established research tools [26].

**Figure 2:**
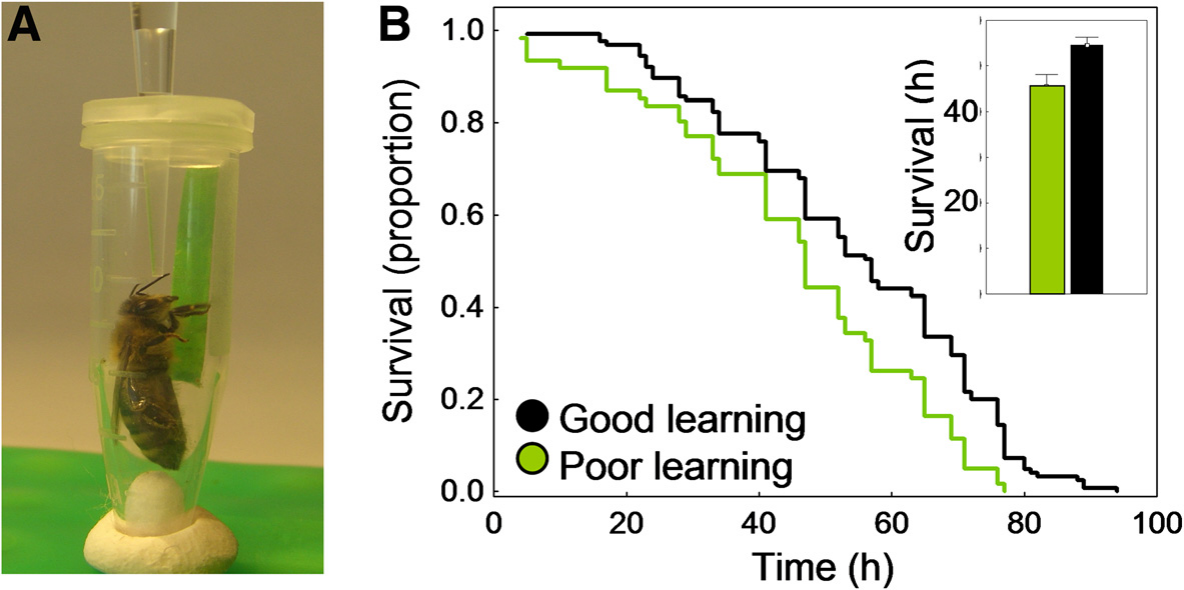
**Panel A: a worker bee in a modified Eppendorf tube that we use in tests of survival capacity in hyperoxia.** The lid has holes for feeding and air exchange. The end of the tube is cut open and sealed with cotton to absorb faeces. Panel **B**: survival data for nurse bees (caregivers) scored as poor learners (green line/bar, learning score = 0 to 3, n = 50) and as good learners (black line/bar, score = 5, n = 85). Nurse bees were collected from four single-cohort colonies; tested over four weeks as independent replicates. Replicate did not influence the proportion of bees surviving, but learning ability had a significant effect [31].

We recently showed that a measure of brain function, Pavlovian (associative) learning ability [30], is positively correlated with metabolic stress resilience (the ability to recover from a stressful event) in hyperoxia (80% O_2_, experimental paradigm for artificial ageing) measured as survival capacity in worker bees [31] (Figure 2). We quantified learning by training restrained worker bees to pairings of a flower odour (the conditioned stimulus, CS) and a sucrose reward (the unconditioned stimulus, US). After six CS-US pairings, learning could be calculated on a scale from zero to five: 0 = the bee fails to express a learned behaviour; 1 = the bee shows learning one time; 2 = the bee shows learning twice, and so on up to 5 = the bee learns the association in the first pairing, and shows the learned response in all remaining five trials. Reward learning is recorded when the bee extends her proboscis (tongue) to the CS alone, before the US is presented [22]. This learning score correlated positively with the bees’ subsequent ability to survive a stressful solitary confinement in the laboratory (Figure 2). Although similarities in the performances of different animal species need not reflect common functional principles [32], the relationship between cognition and survival in the bee seems to resemble the relationship between high IQ and longevity in humans [31].

How can correlations between honey bee learning ability and survival be explained? Worker honey bees are helper females that have no social hierarchy or differential status among them as long as a queen is present in the colony [33]. Experience, workload, behaviour, diet and environmental exposures to stress or damage can differ between workers because of inter-individual differences in age and social role, but these aspects were controlled for in the experiment by using workers of similar age and social task [31]. Behaviour, diet, stress and damage are also factors that can influence the sucrose responsiveness of worker bees and thereby their motivation to perform well in Pavlovian learning tests with sucrose reward. Motivation, however, did not covary with longevity in our study and could not explain the correlation of learning performance and the bees’ ability to survive. It is, in other words, unlikely that socio-environmental factors can account for the observed correlation between learning ability and survival in worker honey bees [31]. This conclusion suggests a biological explanation.

Honey bee brain function, as measured by Pavlovian learning ability, correlates with expression of specific proteins in the brain [34]. In young individuals, learning ability is related to proteins involved in neuronal structure (actin-related protein), adenosine triphosphate (ATP) consumption (vacuolar ATPase, adenylate kinase), neuronal function and signaling (a fatty acid binding protein), in addition to metabolism (aldo-keto reductase, cytochrome P450 homolog). The actin-related protein is more abundant in the brains of individuals that perform well in the test, while the remaining proteins are more abundant in individuals that perform poorly. These high levels of metabolically important vacuolar ATPase, adenylate kinase, aldo-keto reductase and cytochrome P450 led us to hypothesize that poor learning ability in honey bees, prior to senescence, is partly explained by metabolic changes in the brain [34]. Brain ageing in worker bees is associated with a decline in signaling kinases (protein kinase C), synaptic (synapsin) and neuronal growth-related proteins (failed axon connections (fax), nervous wreck (Nwk)) in addition to the fatty acid-binding protein that was identified in younger individuals [35]. These results led us to propose that honey bee brain senescence is partly accounted for by changes that include signal transduction deficiencies. Overall, these studies do not provide a candidate mechanism for system integrity, but the possible connections between metabolic changes in the brain, poor learning ability, and reduced survival in honey bees might lend some support to the idea that variation in metabolic robustness plays a general role in correlations between brain performance and longevity.

We obtained additional information by reversing ageing in worker bees [34]. It is possible to alter the trajectory of honey bee senescence because the rate of worker ageing is a function of social role [36,37]. During the initial weeks of life, worker bees tend to the nest and later in life they forage outside for nectar, pollen and water [38]. Nest bees age slowly, while foragers age rapidly (reviewed by [39]), but senesced foragers can revert to nest tasks if the social demography of the colony is altered in such a way that there are too few young bees in the nest [40]. After this task reversion, about 50% of the previous foragers that are nursing improve brain function [34]. This recovery correlates with changes in the brain’s levels of proteins associated with structure (tubulin alpha-3), stress response/cellular maintenance processes (heat shock protein 8, peroxiredoxin 6), and with neuronal function and signaling (a glutamate transporter most similar to vertebrate excitatory amino acid transporter 2, (EAAT2)) [34]. These data suggested to us that recovery-related brain plasticity is connected to cellular stress resilience, maintenance and repair processes in honey bees. The study might provide examples of functional reserve capacity as well as metabolic robustness, but the lifespan implications of the bees’ recovery are unknown because their longevity was not monitored in the experiment.

At the level of behaviour and longevity, the effects of laboratory handling and social isolation have been investigated in honey bees. Laboratory handling results in life-shortening stress [41] and social isolation has similar negative outcomes that include changes in hormonal axes and the brain [42,43]. These results indicate that our cognition-survival experiment provided stressful test conditions: a Pavlovian learning test in restraints, and a survival test in social isolation. It is unclear how stress affects honey bee learning ability in the laboratory, but stress can depress the levels of dopamine and octopamine in the bee brain and have a negative impact on reward-seeking behaviour in free-flight experiments [44]. If we can assume that stress also reduces a bee’s learning performance in the laboratory, and similarly reduces survival in social isolation, then it is reasonable to ask whether (co)variation between behavioural and molecular mechanisms of coping with stress explains the cognition-survival correlation that we observed [34].

### Stress resilience as a proxy for systems integrity?

**Figure 3:**
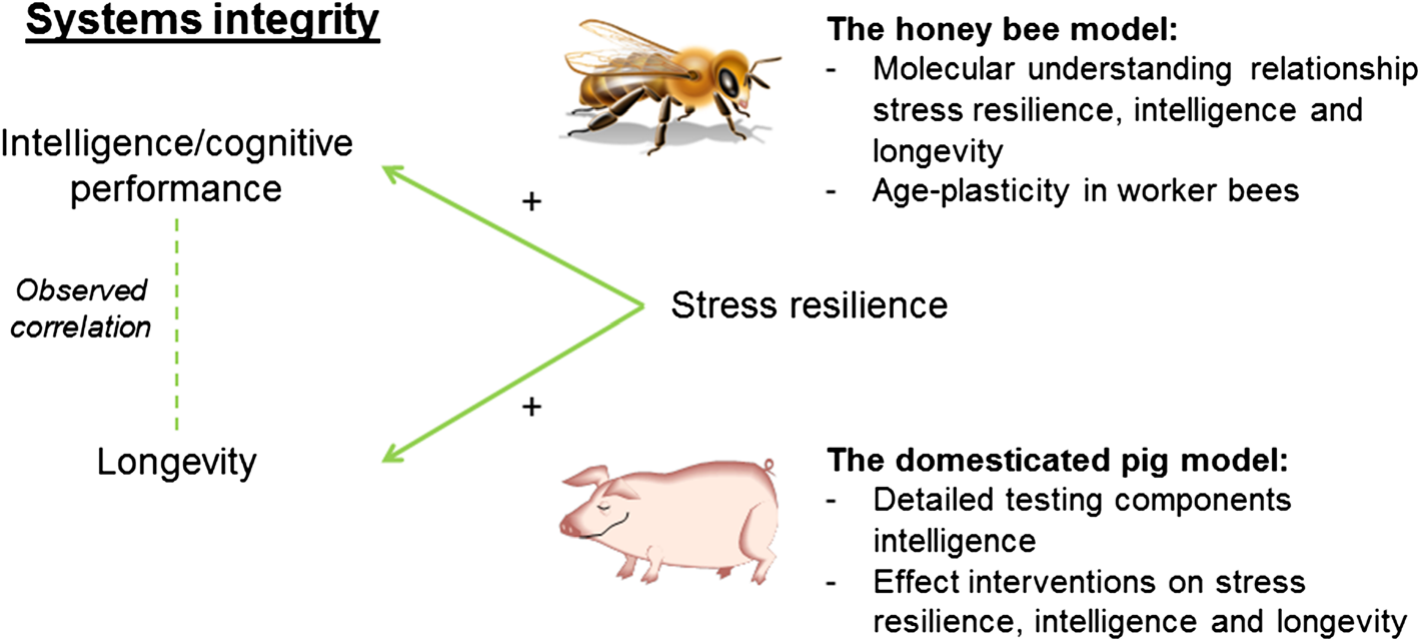
Proposed relationship between stress-resilience, intelligence and longevity, and the potential roles of the honey bee and the domesticated pig as models for systems integrity research.

Stress resilience may thus be a mediating factor influencing the relationship between longevity and brain function (Figure 3). In the context of systems integrity, it is important to distinguish between acute and chronic stress, as these may have different effects on health. Experiencing immediate stress is usually helpful to the individual and results in an adaptive behavioural and physiological response for that situation, the so-called allostatic response. The allostatic response is a physiological response to deal with disturbances in the internal and external environment and return to allostasis, which is in mammals accompanied by an activation of the sympathetic-adrenal-medullary (SAM) and hypothalamic-pituitary-adrenal (HPA) axes [45]. When the animal is repeatedly exposed to immediate stressors, or when exposure to the immediate stressor continues and chronic stress is developed, the body constantly has to deal with disruptors that require an allostatic response, which results in an increase in allostatic load (the cumulative damage that is caused by allostatic responses). This increase in allostatic load can eventually lead to allostatic overload and to several stress-related dysregulations of metabolism, immune function and the brain that can detrimentally affect longevity [46]. Individual variation in stress resilience and its relation to variation in health, behaviour and physiology has been a topic of interest for several decades, in various contexts and in various species. Some of these species may be a complimentary animal model to the honey bee for research on systems integrity.

### The pig: an unusual, but potentially useful model for systems integrity

The pig, with its maximum life span of 27 years [47] is not a very typical model animal in ageing research. Papers that mention old pigs (usually the females, sows) use age in a reproductive setting: a domesticated sow in animal husbandry is labeled as old when her reproductive performance decreases, which is usually around the fourth year of life [48]. Pigs have, however, showed their merit as model animals for neurobiological, physiological and behavioural research. The close resemblance between pigs and humans in terms of genes, brain and physiology makes translation of research findings to humans easier than findings from, for example, rodent research [49-52]. Due to the practical and ethical issues surrounding primate research, the pig has become a more popular animal model, for example in the fields of surgery, pediatric research and neuroscience see [51] for review, [53].

Although not a typical model for ageing, the pig is an interesting model for studying the relationship between cognition, stress resilience and age-related parameters. There is a large knowledge base regarding mechanisms for coping with stress, acute and chronic stress physiology, behaviour and cognition of pigs and a long history in research, although a number of those studies were initiated with commercial goals in mind rather than basic research.

Most of the knowledge on stress physiology and coping with stressful situations has been gathered with animal welfare and animal production in mind, starting in the 1980s. Intensification of animal husbandry in Europe, the United States, Canada and Australia was at its highest, and animal welfare in intensive husbandry became of interest. The early research focused mostly on the optimization of housing conditions to the needs of the animal for example [54,55]. Growth and reproductive performance were important parameters, but also the reduction of behavioural problems (damaging of other animals and stereotypies such as sham chewing) was of great interest. This emphasis resulted in a number of studies that aimed to identify causes of seemingly maladaptive stereotypic behaviours that were thought to indicate a failure of coping with the situation, for example [56,57]. One intriguing insight from the experiments was that performing stereotypic behaviour seems to reduce stress in pigs performing them, suggesting that performing stereotypic behaviours actually help pigs to cope with stress during the performance, although the need for performing stereotypic behaviours still indicates compromised welfare [58,59]. Not all pigs, however, show stereotypic behaviour, implying individual variation in stress resilience and in strategies for coping with stress.

Indeed, different coping strategies have been identified in the pig, which result in different behavioural and physiological responses to both immediate and chronic stressors although for critique see [60,61]. Variation in the response to a stressor has been shown in a wide variety of other species, including mammals, birds, reptiles and insects, and has been described as coping styles, animal temperament, behavioural syndromes and animal personality [62], which typically is described in two different classes: active versus passive, proactive versus reactive, shy versus bold, and so on. The test used to identify different coping styles in pigs is the back test, in which the (young) pig is restrained on its back, which should elicit an anti-predator response [63]. Some pigs will struggle and vocalize (active coping style), while other pigs will remain still and silent during the one-minute test (passive coping style). This response to an immediate stressor (the researcher posing as a predator) has been linked to variation in aggressive and playful behaviours [64], fighting strategy [65], immune response [66], cortisol response in novel situations and restraint [67], heart rate [68], fat and energy metabolism [69], vulnerability for developing gastric lesions, stereotypic behaviour [68] and response to social isolation [70]. The behavioural aspect of coping styles is particularly visible during immediate stress or at the beginning of the chronic stress, when the animals show their strategy to cope with the stressor by trying to regain control of the situation or by quickly adapting to the situation. Although animals of both coping styles may mount a stress response in unfamiliar or risky situations, it is the animals with an active coping style that adapt less quickly to challenging environments and may have lower stress resilience [67,71].

Coping style, as a correlate of stress resilience, is implicated in variation in cognitive performance in pigs. This may be due to a number of different factors. One factor is the response to handling and to the test situation. If the animals are habituated to the test and being handled, acute stress is less likely to affect learning performance. Some cognitive performance tests, however, can elicit a stress response from the animal that can affect their learning performance. Acute stress is implicated in enhanced memory of the event occurring during and after the stress while chronic stress is implicated in reduced learning and memory [72]. In rats, the animals that showed high levels of activity in a novel environment (active coping style) showed a reduction in cognitive performance after chronic stress, supporting the hypothesis that variation in stress resilience is linked to variation in cognitive performance, as is also seen in honey bees [73]. Habituation to the test environment and low-stress housing can reduce stress-induced variation in cognitive performance. This does, however, not eliminate the coping style differences in cognitive performance [74]. This is particularly true when the test setup allows for different strategies of learning or requires a certain amount of behavioural flexibility.

### The pig toolbox: from testing cognitive performance to interventions

**Figure 4:**
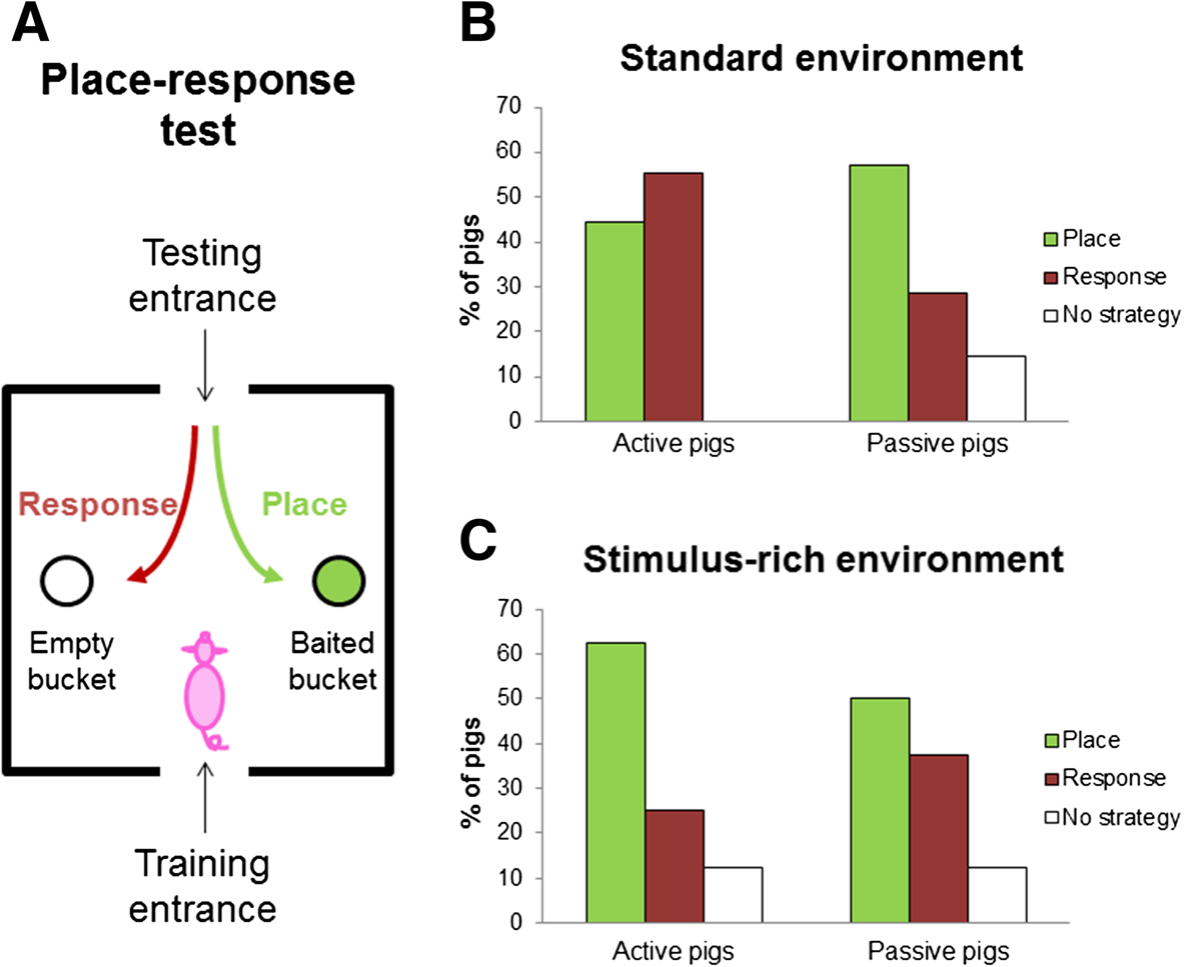
**Panel A: overview of the place-response test.** Pigs approached buckets from one entrance during training, learning to find a baited bucket. Pigs needed to find the treat in the baited bucket without investigating the empty bucket for five trials in a row before proceeding to the test phase. In the test phase, the pig approached the buckets from the opposite site of the arena. If the pig had learned the place of the baited bucket, it would go to the baited bucket; if it had learned to turn towards the right on entering (response), it would visit the empty bucket. Panel **B**: Active pigs that were housed in a standard, stimulus-poor environment (barren, no substrates provided) from birth until testing (eight weeks of age) showed response learning more often than passive pigs. Panel **C**: Active and passive pigs have similar strategies when they have been housed in a stimulus-rich environment (enriched, more complex environment with substrates for exploration provided) from birth onwards.

An example of a learning test that requires behavioural flexibility is given in Figure 4. The place-response test is a simple test of association of a place with a reward (panel A). Pigs will learn that one of two buckets is baited on either the right or left side of the test arena. After the association is successfully learned, the pig is brought into the arena from the opposite entrance. If the pig has learned the place of the baited bucket (place learning) then it will visit the correct bucket. However, if the pig has simply learned to turn in one direction after entering the arena (response learning), it will visit the unbaited bucket. Panel B shows that pigs with an active coping style tend to show response learning more often than pigs with a passive coping style. Differences in information use (attention to and memory of cues in the environment), as well as behavioural flexibility may play a role in these coping style differences. A similar coping style difference was found in a T-shaped maze, where active coping pigs had more difficulties learning to change direction to find the baited bucket [75]. Other factors that may be related to variation in performance in cognitive testing are reward sensitivity and behavioural inhibition [76,77], though those have not been explored in detail in pigs.

Pigs can be used in different types of learning tasks, which makes them very useful models for cognitive testing. Pigs can be trained to operate levers, joysticks handles and rotating wheels for operant conditioning. Other learning paradigms that have been used in pigs include classical conditioning, social recognition tasks, spatial learning and memory tasks, and observational learning tasks [78-80]. The above mentioned tests measure different aspects related to cognitive performance and intelligence. The hole-board test, for example, tests both reference memory (the place where the rewards can be found) and working memory (the ability to temporary store and manage information, such as which buckets were already visited during the trial) [81]. Reference memory and working memory seem to be two uncorrelated systems, and particularly working memory is linked to general intelligence in humans [82]. While a honey bee can solve computational problems that include rule learning, non-elemental learning, delayed matching-to-sample tasks, categorization and numerical processing of numbers up to and including four, she is limited in the type of cognitive performance tasks that can be used compared to the pig reviewed by [83]. Thus, the pig can be used as a model for a more in-detail testing of the link between different aspects of memory and ‘intelligence’, stress resilience and ageing-related parameters. Besides learning tasks there are a range of neurobiological, stress-physiological, metabolic and molecular tools available that are highly relevant for this research. These tools include *in vivo* PET-scans [84], non-invasive measurement of salivary stress hormones such as cortisol [85], measuring energy expenditure using indirect calorimetry in metabolic chambers [71] and measuring biomarkers of ageing such as oxidative stress and telomere length [86,87], which are complimentary to the tools available in less complex systems such as the honey bee. Finally, it is also possible to control the environment in which the pig develops. This can be done already before birth, by various behavioural, dietary and reproductive interventions for example [88-90], as well as after birth. This control of the environment allows for studying the effects of environmental factors. For example, increased interactions with the mother early in life (‘education’) resulted in a better ability to cope with the stress associated with early weaning in pigs in commercial husbandry, as well as in the ability to cope with the switch from a high-quality environment to a low-quality environment [91].

Environmental factors may affect stress resilience and cognitive performance, but whether interventions targeting these environmental factors will also affect longevity is unknown. An interesting model approach is the use of environmental enrichment. Enriching the environment by increasing space allowance, allowing for more interactions with conspecifics or by providing enrichment materials has profound effects on brain development, brain ageing, cognitive performance and stress resilience [92-95]. In the pig, environmental enrichment early in life may reduce differences in behavioural flexibility and cognitive performance between animals with different levels of stress resilience, as seen in Figure 4C. Environmental enrichment can be used as a model in pigs to investigate links between cognitive performance, stress resilience and health/longevity: do interventions that improve of one of these factors also improve the other factors? If this is the case, then one can ask the question how flexible the links between the different factors are later in life, and whether enrichment as a model intervention later in life can negate the built-up allostatic load and the increased risk for allostatic overload and dysregulations that can negatively affect longevity. Understanding whether the different traits of systems integrity are sensitive for interventions early and later in life will be the first step in exploring options for interventions that target human health and lifespan.

### Human beings: evidence for the importance of stress resilience in cognitive performance and longevity

So is there any evidence that stress resilience and the ability to cope with stress are implicated in the relationship between intelligence and longevity in humans? Coping style in the pig is a way of describing animal personality, and is, like personality measures in other non-human species, measured on one axis: the behavioural response to a stressor. Human personality, on the other hand is typically a construct of multiple axes. Famous examples include the Big Five, Myers-Briggs indicators and type A or B personality [96-98]. When translating findings in animal research to humans it may be important to look specifically at the behaviours measured when determining animal personality and specific elements of the human personality constructs. We speculate that some specific elements of human personality that may be important in the context of stress resilience and systems integrity are hostility (type A personality), optimism, neuroticism (Big Five, also measured as impatience and time-urgency in Type A personality), openness (Big Five) and conscientiousness (Big Five).

Although personality is not directly linked to measures of IQ, there are indications that personality affects performance in cognitive testing in humans. Anxiety (measured in neuroticism) is an obvious candidate in the context of systems integrity. Indeed, increased levels of anxiety (and stress) tend to shift attention away from the cognitive task and towards threat-related stimuli [99], thereby reducing performance. Impulsivity and the lack of behavioural inhibition (implicated in hostility) can also interfere with performance in cognitive tasks, depending on the nature of the task [100]. Conscientiousness and openness are implicated in variation in cognitive performance as well, though the results are ambiguous and may depend on the type of measurement or test [101-103].

There are, furthermore, indications that stress resilience and personality characteristics are connected to longevity. In general, psychological stress and metabolic stress are implicated in increased cellular stress, reduced telomerase activity and telomere shortening [104]. Psychological stress can reduce immune function, thus increasing susceptibility for disease [105]. It may thus pay off to be less sensitive to stress, or to avoid stressful situations. Some specific personality characteristics may affect the sensitivity to stress and longevity. Hostility is positively correlated to artery blockage [106], which in turn can reduce longevity. Optimism is related to reduced longevity [107], particularly early in life. A study in Tokyo centenarians showed that type B personality was more prevalent in centenarians than in 60-year-olds [108]. A Japanese cohort study found positive relationships between conscientiousness, extravertedness, openness and longevity [109]. These traits were found to be dominant in the Tokyo centenarians as well [110]. In addition, Georgia centenarians also showed low levels of neuroticism [111]. Of course, part of the above mentioned findings can be caused by differences in health behaviours, particularly those related to conscientiousness, but it would be worth revisiting these findings in the context of systems integrity.

## Conclusion

The concept of systems integrity and the potential role of stress resilience in determining both cognitive performance and longevity need to be explored further. A recent paper [112] describes the prerequisites for supporting evidence for system integrity. The first is that there is a plausible marker trait for the latent trait of systems integrity. We propose stress resilience as such a trait. Second, the trait should be correlated with health or longevity traits. This seems to be the case in the honey bee, and there is epidemiological evidence for the same phenomenon from humans, but a more experimental approach should be designed to test the hypothesis. Third, the different traits (such as intelligence and stress coping) should be correlated, preferably early in life. We showed some evidence for this association from human studies, and an experimental approach in pigs that also looks at the flexibility of the system. Testing this in the bee is a logical next step. Finally, there should be covariance between the different traits in the effect on health and longevity. Such information is missing for stress resilience, but can be tested with help of the two model animals proposed in this paper.

The honey bee and the pig each provide experimental advantages that are different and complimentary. The bee is a short-generation animal in which ageing can be reversed, which makes it an ideal subject for longevity studies in the context of systems integrity. Although the pig lives much longer than a bee, it is also phylogenetically and physiologically closer to humans, and provides an interesting model for further exploration of stress physiology, cognition and longevity and of the effects of interventions early and later in life. As the person’s average life has become more stressful in the past 30 years [113], and the number of elderly people increases, the question of how to get old successfully is one of interest to everyone.

## Competing interests

The authors declare that they have no competing interests.

## Authors’ contribution

MO participated in the design and carrying out of the place response test and drafted the manuscript. GVA participated in the design and carrying out of the bee learning and survival tests and helped to draft the manuscript. All authors read and approved the final manuscript.
